# Shear-Stress Sensitive Inwardly-Rectifying K^+^ Channels Regulate Developmental Retinal Angiogenesis by Vessel Regression

**DOI:** 10.33594/000000109

**Published:** 2019

**Authors:** Evgenii Boriushkin, Ibra S. Fancher, Irena Levitan

**Affiliations:** a Department of Medicine, Stony Brook University, Stony Brook, NY, USA,; b Division of Pulmonary, Critical Care, Sleep and Allergy, Department of Medicine, University of Illinois at Chicago, Chicago, IL, USA

**Keywords:** Angiogenesis, Shear stress, Potassium channels, Endothelium

## Abstract

**Background/Aims:**

Shear stress plays major roles in developmental angiogenesis, particularly in blood vessel remodeling and maturation but little is known about the shear stress sensors involved in this process. Our recent study identified endothelial Kir2.1 channels as major contributors to flow-induced vasodilation, a hallmark of the endothelial flow response. The goal of this study is to establish the role of Kir2.1 in the regulation of retinal angiogenesis.

**Methods:**

The retina of newly born Kir2.1^+/−^ mice were used to investigate the sprouting angiogenesis and remodeling of newly formed branched vessels. The structure, blood density and mural cell coverage have been evaluated by immunohistochemistry of the whole-mount retina. Endothelial cell alignment was assessed using CD31 staining. The experiments with flow-induced vasodilation were used to study the cerebrovascular response to flow.

**Results:**

Using Kir2.1-deficient mice, we show that the retinas of Kir2.1^+/−^ mice have higher vessel density, increased lengths and increased number of the branching points, as compared to WT littermates. In contrast, the coverage by αSMA is decreased in Kir2.1^+/−^ mice while pericyte coverage does not change. Furthermore, to determine whether deficiency of Kir2.1 affects vessel pruning, we discriminated between intact and degraded vessels or “empty matrix sleeves” and found a significant reduction in the number of empty sleeves on the peripheral part of the retina or “angiogenic front” in Kir2.1^+/−^ mice. We also show that Kir2.1 deficiency results in decreased endothelial alignment in retinal endothelium and impaired flow-induced vasodilation of cerebral arteries, verifying the involvement of Kir2.1 in shear-stress sensing in retina and cerebral circulation.

**Conclusion:**

This study shows that shear-stress sensitive Kir2.1 channels play an important role in pruning of excess vessels and vascular remodeling during retinal angiogenesis. We propose that Kir2.1 mediates the effect of shear stress on vessel maturation.

## Introduction

The formation of a functional network of blood vessels is essential for vertebrate development and organ function. New blood vessel formation is called angiogenesis and consists of two major phases: first is sprouting, the process of forming new vessels to create a meshwork of branched capillaries, and then remodeling, the process of removal or pruning of redundant vessels to form a mature plexus and to increase the efficiency of the perfused network [1, 2]. Both phases of angiogenesis have been extensively studied and the mechanisms of sprouting are well understood [3, 4] but regulation of the remodeling process is less clear. Several studies showed that one of the important remodeling factors in developmental angiogenesis is shear stress, a frictional force generated by blood flow, that is known to play a major role in vascular remodeling in general [5]. Furthermore, studies in several developmental models, including zebrafish, and murine yolk sac, showed that shear stress is required for the remodeling but not for endothelial sprouting and vessel branching [6–9]. As soon as new vessels have a lumen and blood starts to flow, endothelial cells become exposed to shear stress, which stabilizes the vessel and induces recruitment of mural cells and deposition of extracellular matrix into the basement membrane, whereas vessels with no or low flow regress. Specifically, early studies in Zebrafish developmental model showed that while flow does not have a major effect on the formation and patterning of the primary vascular network, vessels with little or no blood flow tend to undergo regression [6]. Furthermore, Lucitti et al. demonstrated that vascular remodeling in a murine yolk sac requires mechanical force generated by the blood flow [7]. They also demonstrated that, similarly to the well-known effects of shear stress on the morphology of endothelial cells and expression of endothelial nitric oxide synthase (eNOS) *in vitro* [5], shear stress results in endothelial elongation and an increase in eNOS expression in the arteries of murine yolk sac [7]. More recently, Franco et al. [8] demonstrated that regression of blood vessels in mouse retina results from flow-sensitive migration of endothelial cells from vessels with low flow to vessels with higher flow and thus results in the maturation of the plexus. It is important, therefore, to understand the mechanisms that might couple between shear stress and developmental vascular regression.

Our recent study [10] discovered that flow-induced activation of eNOS critically depends on a specific type of endothelial K^+^ channel, inwardly-rectifying Kir2.1. In general, inwardly-rectifying K^+^ (Kir) channels are a major class of K^+^ channels expressed in multiple cells and tissues [11, 12]. The major function of Kir channels is regulating cell membrane potential with activation of the channels leading to K^+^ efflux and membrane hyperpolarization, which in turn regulates membrane excitability and the influx of calcium [13–15]. Kir channels are classified into seven sub-families (Kir1–7) differentially expressed in different tissues and sensitive to different stimulators (reviewed by [13, 15]. The sub-family of Kir2 channels is the most ubiquitously expressed and known to be involved in excitability and contraction of cardiac and smooth muscle cells [16–18], neurovascular coupling in cerebral arterioles [19] and propagation of hyperpolarization in capillary endothelial cells [20]. Kir2.1 channels have long been known to be expressed in endothelial cells (ECs), both mature and progenitor ECs [21–23] including in retinal endothelium [24]. Endothelial Kir channels were also shown to be sensitive to fluid shear stress generated by flow and it was proposed that they constitute a primary shear stress sensor in endothelial cells [22, 25, 26]. Our recent studies provided the first compelling evidence to support this hypothesis by demonstrating that endothelial Kir2.1 channels are essential for flow-induced activation of eNOS and release of NO in microvascular endothelial cells from the mesenteric vascular bed [10]. Notably, it is well known that flow-induced vasodilation is mediated by the combination of NO-dependent and NO-independent pathways, the latter mediated by the endothelium-dependent hyperpolarizing factor (EDHF), which most studies identify as small and/or intermediate-conductance Ca^2+^-sensitive K^+^ channels. Indeed, we showed that the contributions of Kir2.1 and of Ca^2+^-sensitive K^+^ channels to flow-induced vasodilation are additive with Kir2.1 mediating the vasodilation via an NO-dependent mechanism [10]. These studies established Kir2.1 as a key element of shear stress-induced endothelial signaling.

In this study, therefore, we addressed the question of whether Kir2.1 channels play a role in developmental angiogenesis using the mouse retina as a well-characterized and robust tool for *in vivo* developmental angiogenesis [27, 28]. We show here that deficiency in Kir2.1 channels compromises the alignment of endothelial cells in the vessels of developing retina and impairs cerebrovascular response to flow, indicating that these channels play an important role in flow mechanotransduction in the retina and cerebral microcirculation. Most importantly, we show that Kir2.1 deficiency results in impaired vascular remodeling in the retinal vascular plexus.

## Materials and Methods

### Mice

Kir2.1^+/−^ mice on FVB background were obtained from JAX and bred in our laboratory. This model is used because Kir2.1^−/−^ mice die within hours after birth [18] whereas Kir2.1^+/−^ are fully viable. Retinal phenotypes of mutant mice were analyzed at postnatal day 6 (P6). All animals and protocols were used in accordance with the Institutional Animal Care and Use Committee of University of Illinois at Chicago, Case Western Reserve University and the National Institutes of Health Guide for the Care and Use of Laboratory Animals.

### Processing and analysis of retinas

Dissection and whole mount staining of retinas were performed as previously described [28]. Enucleated eyes were fixed for 1 h in 4% paraformaldehyde, rinsed three times in PBS, dissected and stored in methanol at −20°C. Immunohistochemistry of whole-mount samples was performed by using isolectin IB4 (Iso B4; Thermo Fisher Scientific, catalog #I21411, 1:200), rabbit anti-desmin antibody (Abcam, catalog #ab8592, 1:100), mouse anti- α-SMA (Sigma, catalog #F3777, 1:100), rabbit anti-collagen IV (Abcam, catalog #ab6586, 1:100) and rabbit anti-CD31 (Abcam, catalog # ab28364, 1:100). For detection, suitable specific Alexa Fluor-coupled secondary antibodies were used (Thermo Fisher Scientific, 1:1000). For the *in vivo* BrdU incorporation assay, 100 μg of BrdU (BD Pharmingen) per gram of body weight was injected intraperitoneally 4 h before the mice were euthanized with CO_2_ followed by cervical dislocation. Retinas were isolated and collected for analysis as above. BrdU positive cells were stained by mouse anti-BrdU antibody (Cell signaling, catalog #5292, 1:100).

### Microscopy and image analysis

Fluorescent images were taken using a Leica DM2000 microscope for low-magnification images and a Leica Sp8 confocal laser scanning microscope for high magnification images. The images were processed using ImageJ (NIH, Bethesda, MD) and vascular characteristics of the retina were analyzed using Angiotool software (National Cancer Institute) [29]. Quantification is based on a minimum of six mutant and six control animals for each time point and experimental condition, and mice were litter-matched. For phenotypic analysis, low-magnification images were taken of isolectin B4-labeled control and mutant retinas. A composite picture of the whole retinal vasculature was obtained from partially overlapping images (×10 lens) by using Photoshop CS5 (Adobe Systems) as shown previously [28]. Quantitation of vessels density was performed in each quadrant of the whole-mount on the vascular plexus located between an artery and a vein. Quantitation of branching points and total vessel length was performed using the entire composite picture of the retina. For quantitation of sprouts and filopodia, confocal images of the angiogenic front were used. The angiogenic front was defined as the line connecting the bases of the sprouting ECs. The total number of filopodia was counted per angiogenic front in each field and calculated as the ratio of the total number of filopodia at the angiogenic front line per field (250μm). Arteries and veins were identified based on standard morphological criteria. The total length of α-SMA positive arteries was measured in each retina from the optical nerve head in the central retina to the end of the α-SMA positive portion of the arteries. Pericyte coverage was quantified as the desmin-positive area among the total isolectin B4-positive area. To analyze vessel remodeling, the whole-mount retinas were labeled with isolectin B4 and collagen IV. A set of high-resolution images of the central capillary plexus between an artery and a vein was taken and quantified for the ratio of collagen IV positive to isolectin B4 negative sprouts.

### Quantification of endothelial cells alignment

Using the images with CD31 staining of retinal arteries from optic nerve to angiogenic front at P6, we quantified the endothelial cell alignment in the direction of flow by measuring the angle between the long axis of the cell determined visually and the axis of the vessel using MetaVue 6.2R6 software (Molecular Devices Inc). Cells that are aligned perfectly in the direction of the flow (angle coincide with the axis of the vessel) have an angle of 0° and the full range of angles theoretically can vary between −90 and 90°.

### Flow-induced vasodilation in middle cerebral arteries

Brains of 20 week old WT and Kir2.1^+/−^ mice were excised and immediately placed in ice cold HEPES buffer (containing in mM: 140 NaCl, 4 KCl, 1 MgCl_2_, 5 glucose, 10 HEPES, 2 CalCl_2_, pH 7.40). Middle cerebral arteries (MCAs) were removed, cleaned of connective and parenchymal tissue, and cannulated in specialized chambers designed for video microscopy. Continuously circulated Krebs buffer (in mM: 123 NaCl, 4.7 KCl, 1.2 MgSO_4_, 2.5 CaCl_2_, 16 NaHCO_3_, 0.026 EDTA, 11 glucose, 1.2 KH_2_PO_4_, pH 7.4, 37°C) was used for experiments. Prior to analysis of dilations to flow, MCAs were pressurized at 60 cm H_2_O for 1 hour by two Krebs filled reservoirs which perfuse the artery from either side. After preconsriction with endothlin-1 to 50% of the baseline diameter, intraluminal flow was administered by raising one reservoir while simultaneously lowering the other an equal distance in a dose-dependent fashion. This method generates a pressure gradient that allows for increases in intraluminal fluid flow with minimal changes in intraluminal pressure to isolate the effects of shear stress on the vascular wall [10, 30, 31]. Changes in diameter were recorded using a VIA-100 Boekeler calibrated for horizontal measurements. BaCl_2_ (30 μM) was added to the circulating bath and incubated for 30 minutes prior to repeating the protocol. Arteries that did not constrict at least 50% to 200 pM ET-1 were discarded from study. Furthermore, papaverine (100 μM) is applied at the end of each protocol to ensure vascular smooth muscle function remains intact throughout the experiment. Arteries that did not dilate to papaverine >80% of the baseline diameter at the end of each protocol were also discarded.

### Statistics

All data are reported as the mean ± SD. Student’s two-tailed non-paired t-test and 2-way ANOVA were used to determine the statistical significance. The significance level was set at p < 0.05 and notated by an asterisk (*).

## Results

### Kir2.1 deficiency increases the length, density and branching of the vascular network in developing retina

**Fig. 1.**
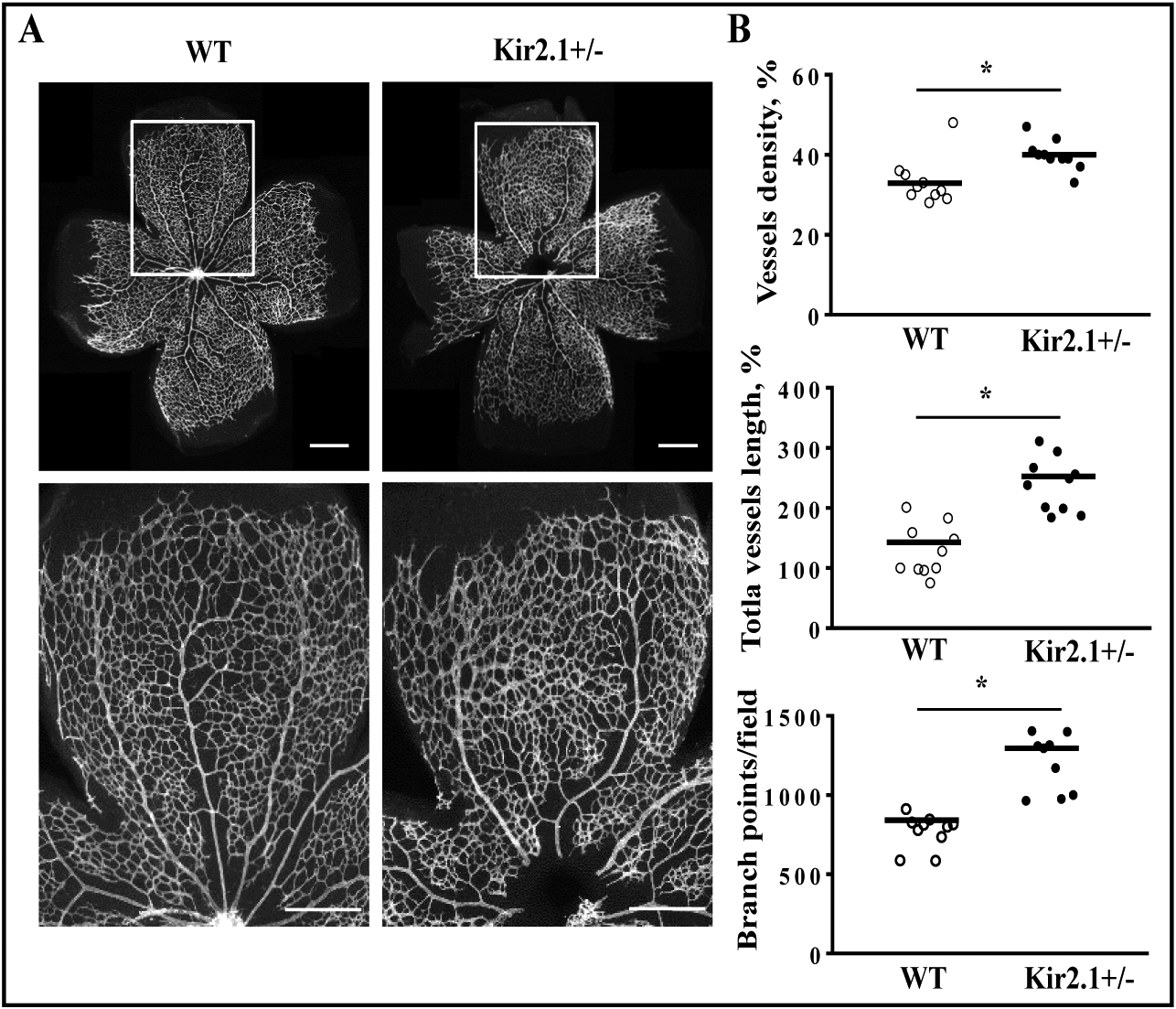
Deficiency of Kir2.1 results in increased vessel density during retinal angiogenesis. (A) The images show increased total number of vessels and vessels density in P6 whole mount retinas in Kir2.1^+/−^ mice (IsoB4, white). The angiogenic front inset from the upper panel is shown enlarged in the lower panel. (B) Quantification of vasculature parameters in wild type and Kir2.1^+/−^ retinas as indicated. Each dot refers to the parameter of an individual mouse retina. Data are means of at least six mice per group. *P≤0.05. Scale bar panel A: 100 μm.

The mouse retina during postnatal days 1–21 (P1–P21) is a unique model of developmental angiogenesis. This angiogenesis model enables the study of the molecular mechanism of both sprouting [32–34] and remodeling of newly formed branched vessels [6–8, 35, 36]. In order to determine if Kir2.1 channels regulate retinal angiogenesis, we examined the structure and density of blood vessel networks in retinas of new born Kir2.1^+/−^ mice. Our recent study showed that Kir2.1^+/−^ mice have significantly decreased expression of Kir2.1 in vascular endothelium in resistance arteries [10]. In this study, the retinas of Kir2.1^+/−^ mice and their WT litter mate controls were examined at day 6 (P6), because at this time point the vessel network covers 75–80% of the retina but does not reach the retina edge. It allows the detailed investigation of both sprouting angiogenesis and vessel remodeling in a tightly controlled setting. Fig. 1A shows representative images of the vascular networks of Kir2.1^+/−^ and WT mice, visualized with Isolectin B4, which binds to the sugar residues of the endothelial glycocalyx and is widely used to visualize blood vessels [37]. The upper panels show the typical structures of the retinas in both types of mice without gross abnormalities. The lower panels, however, which show enlarged angiogenic fronts, suggest a subtle but apparent increase in the network density and branching in Kir2.1^+/−^ mice. Most importantly, quantification of the images show pronounced increases in the lengths of the vessels (Fig. 1B) and the number of the branching points in Kir2.1^+/−^ mice, as well as a less pronounced but still statistically significant increase in vessel density. The average diameters of the vessels used for the analysis range from 5.5 μm in the capillaries to 47 μm in the largest vessels near the optic disc.

**Fig. 2.**
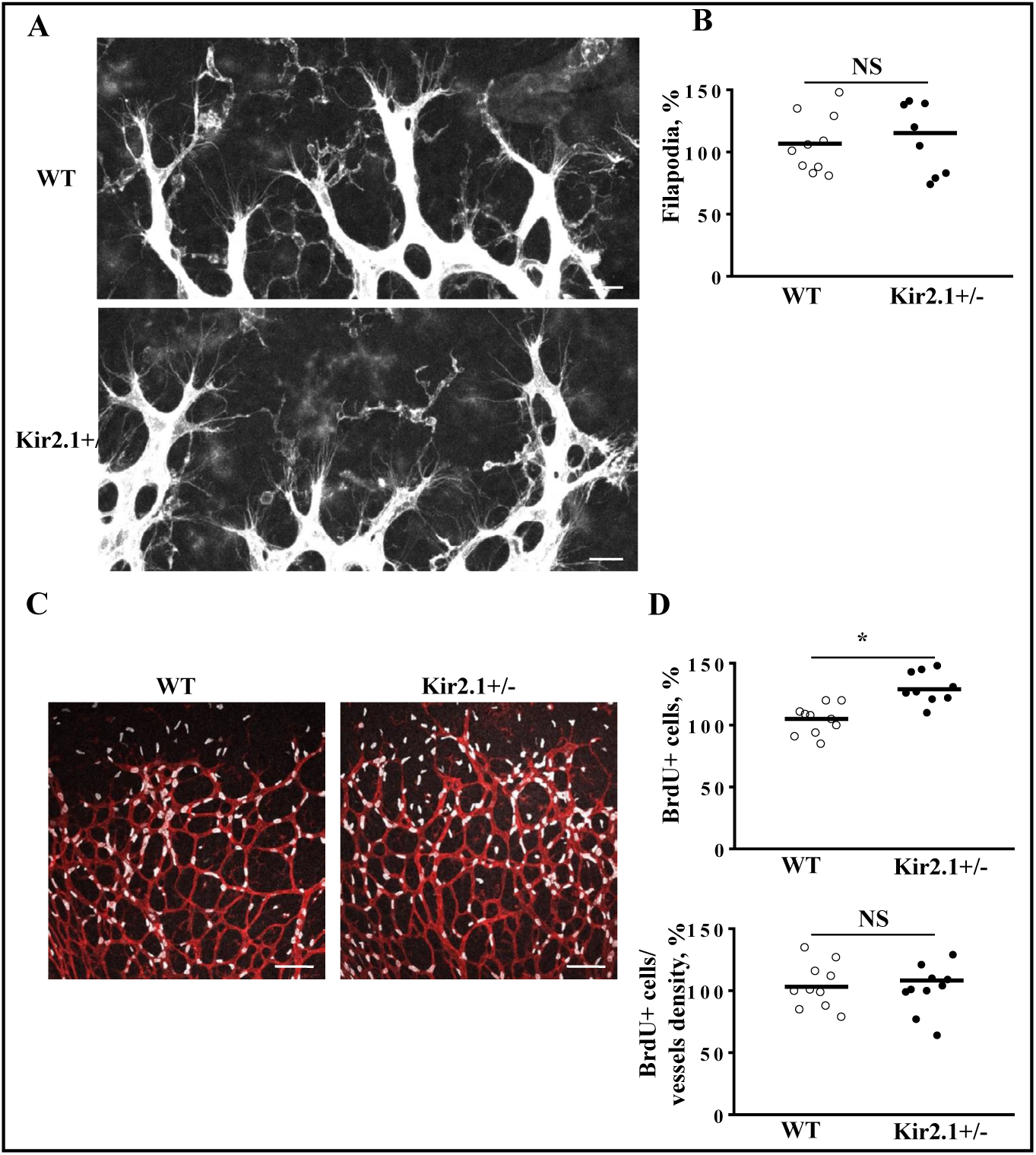
Kir2.1 expression does not alter the number of filopodia and retinal cell proliferation. (A) Images of filapodia of Kir2.1^+/−^ and WT retinas at P6 stained with IsoB4 (white). (B) Quantification of filopodia numbers in WT and Kir2.1^+/−^ retinas as indicated. Each dot refers to the filopodia numbers of an individual mouse retina. (C) Images stained to identify proliferation of the retinal cells by BrdU labeling (white) of WT and Kir2.1 retinas. (D) Upper graph: The total number of BrdU positive cells increased in Kir2.1^+/−^ group. Lower graph: BrdU positive cells normalized to total EC area (isolectin B4, red). Each dot refers to the parameter of an individual mouse retina. Data are means of at least six mice per group. *P≤0.05. “NS” means no significant differences. Scale bar panel A: 25 μm, panel C: 100 μm.

Next, we examined whether an increase in the network parameters described above could be attributed to the enhanced sprouting of the endothelial cells. To this end, we explored and counted the filopodia, the key criteria of the sprouting [38]. However, no difference was found between Kir2.1^+/−^ and WT mice, neither in the appearance, nor in the number of the filopodia (Fig. 2A, B). That indicates that endothelial cells in the Kir2.1^+/−^ mouse retina did not acquire an amplified sprouting mode. Another possible underlying mechanism for the increased blood vessels density and branching may be due to increased proliferation of endothelial cells in the sprouts. To explore this possibility, the retinas were labeled *in vivo* with 5-bromodeoxyuridine (BrdU), a marker of cell proliferation. Our results show an increase in the total number of BrdU positive cells in retinas of Kir2.1 group, as compared to WT (Fig. 2C, D) but no difference in the number of BrdU positive cells normalized to the vessel density (Fig. 2D). This observation indicates that increased cell proliferation cannot account for the observed increase in vascular density.

### Kir2.1 deficiency decreases vascular smooth muscle cells coverage

**Fig. 3.**
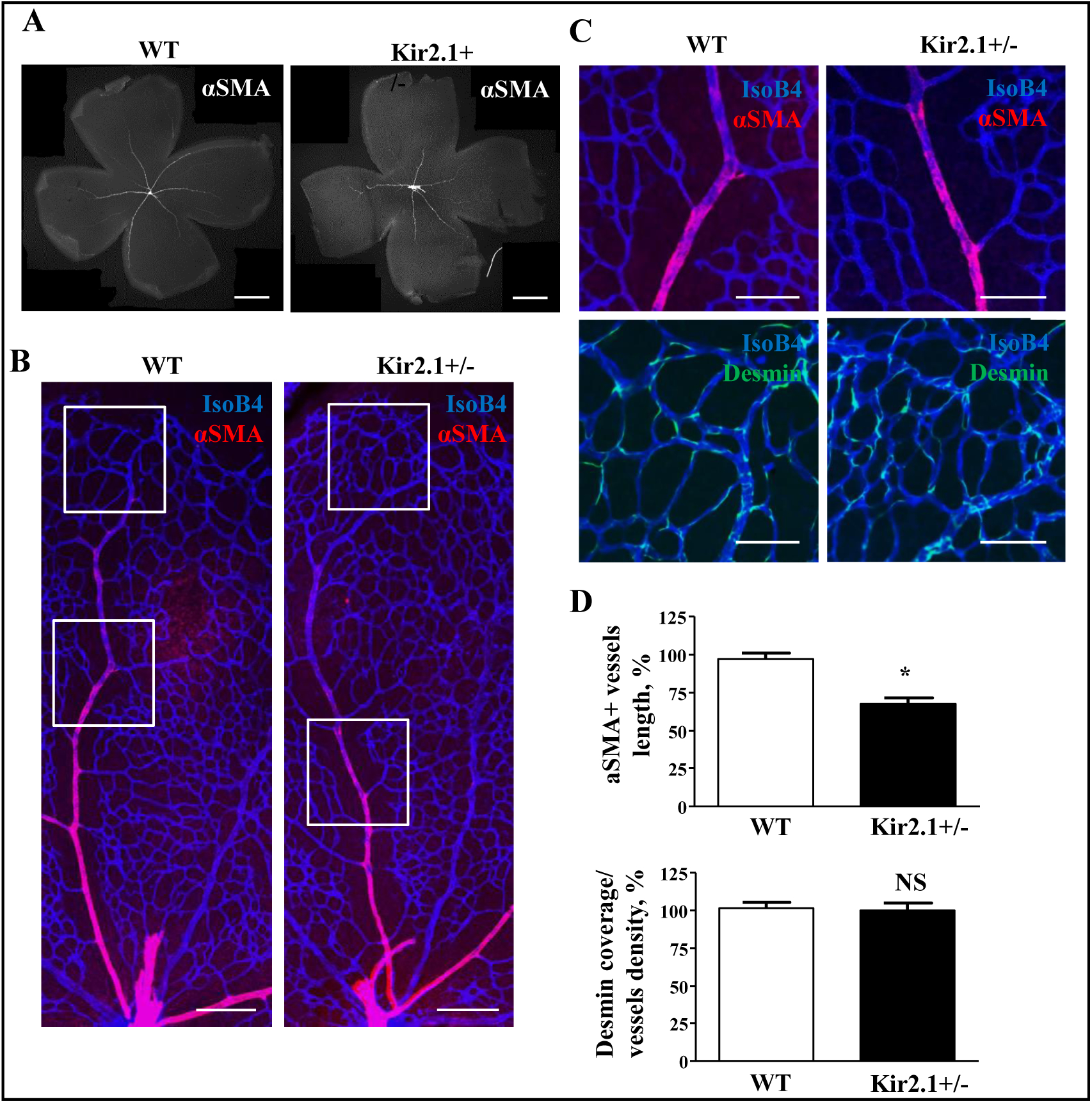
Deficiency of Kir2.1 decreased the smooth muscle cell coverage but does not change pericytes coverage of the retinal vasculature. (A) Mouse retinas were stained with alfa smooth muscle actin (αSMA) to visualize vSMCs. Low magnification images of retina showed decreased the αSMA-positive vessel length in Kir2.1+/− mice compare to wild type. High magnification images of mouse retina stained with αSMA (red) and Isolectin B4 (IsoB4, blue) showed the decreased length of αSMA-covered artery. Panel 3C shows the magnification of the inserts, indicated as squares in Panel 3B. The two upper squares of Panel B are expanded to show Desmin and the two lower squares in Panel B are expanded to show αSMA. Please note that the αSMA region chosen for WT mice is much closer to the periphery of the retina than the region chosen for Kir2.1^+/−^ mice. This is consistent with a decrease in smooth muscle coverage. (D) Quantification of the αSMA and desmin coverage are shown in lower panel. Data are means ± SD of at least six mice per group. *P<0.05. Scale bar panel A,B,C: 100 μm.

Formation of blood vessels also involves recruitment of mural cells, specifically smooth muscle cells (SMCs) identified as the mural cells of arteries, arterioles and veins, and pericytes identified as the mural cells of capillaries [39–41]. As expected, vascular smooth muscle cells were present around the branches of the central retinal artery, as assessed by αSMA staining (Fig. 3A, B), in both Kir2.1^+/−^ and WT retinas (Fig. 3A). However, in contrast to an increase in length, density and branching of the endothelial network in Kir2.1^+/−^ retinas described above, the length of arterial branches in P6 retinas covered by αSMA are decreased in Kir2.1^+/−^ mice (Fig. 3 A, C) suggesting impaired remodeling. The analysis of the pericytes coverage, identified by desmin, showed no difference between Kir2.1^+/−^ and control group when normalized to the total area of the vessels (Fig. 3D), suggesting that endothelium-pericytes interaction is not associated with the vascular alteration seen in Kir2.1^+/−^ group. These data suggest that within the developing retinal arteries, Kir2.1 plays a role in the smooth muscle coverage, but is dispensable for pericytes recruitment during developmental angiogenesis.

### Deficiency in Kir2.1 leads to delay vasculature remodeling

**Fig. 4.**
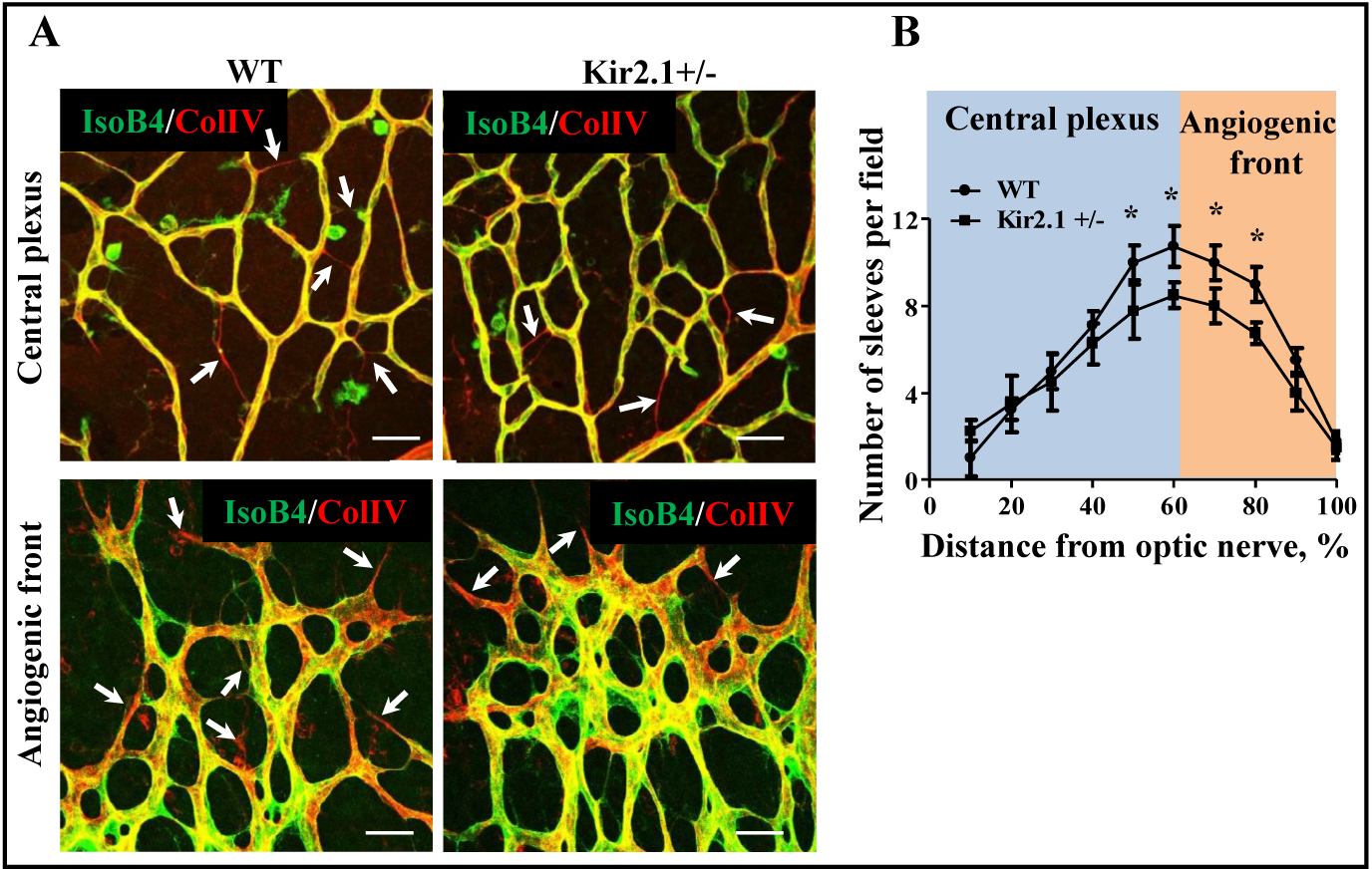
Kir2.1 deficiency leads to delay in vascular remodeling. (A) Images of retinas of WT and Kir2.1^+/−^ mice stained for IsoB4 (green) and collagen IV (red). Collagen positive and isolectin B4 negative indicate empty sleeves that remain after remodeling. (B) Quantification of empty sleeves across the retina in wild type and Kir2.1^+/−^ retina. Data are means ± SD of at least six mice per group. *P≤0.05. Scale bar panel B: 50 μm.

As described in the Introduction, during the first stage of the developmental angiogenesis, the vessel sprouting generates excessive vessels and branching points, which are then pruned during maturation resulting in a highly organized vascular network with less density. It is also known that during the pruning process, the vessel itself is degraded but collagen depositions formed around the vessel remain for at least several days and can be detected as “empty” collagen sleeves [42]. Thus, to determine whether deficiency of Kir2.1 affects the pruning process, we co-stained the retinas for collagen IV using immunostaining and for intact vessels using isolectin B4. This staining clearly detected “empty” matrix sleeves, which are identified as collagen IV positive but isolectin B4 negative [43], in both Kir2.1^+/−^ and WT retinas (Fig. 4A). However, analysis of the spatial distribution of matrix sleeves based on their distance from the optic nerve showed a significant reduction in the number of the sleeves on the peripheral part of the retina, also called the “angiogenic front” in the Kir2.1^+/−^ group (Fig. 4B). These data suggest that Kir2.1 plays a role in vasculature remodeling and pruning during developmental angiogenesis.

### Kir2.1 expression does not affect vessel density in retinas of adult mice

An increase in vessel density described in the previous section was observed only in the developing retinas and did not persist in adult mice (Supplementary Fig. 1 - for all supplemental material see www.cellphysiolbiochem.com). No significant differences were observed in vessels density, vessels length and branching between WT and Kir2.1^+/−^ in 8 week old mice. The lumens of the retinal blood vessels were also similar in WT and Kir2.1^+/−^, which suggests that Kir2.1^+/−^ blood vessels are fully perfused. It is important to note that it is not uncommon to see that differences in retinal angiogenesis during development do not persist in adult mice. The functional significance of these observations is that impairment of retinal angiogenesis is considered indicative of a general defect in angiogenesis.

### Kir2.1 is required for endothelial cell alignment in arteries of developing retinas

**Fig. 5.**
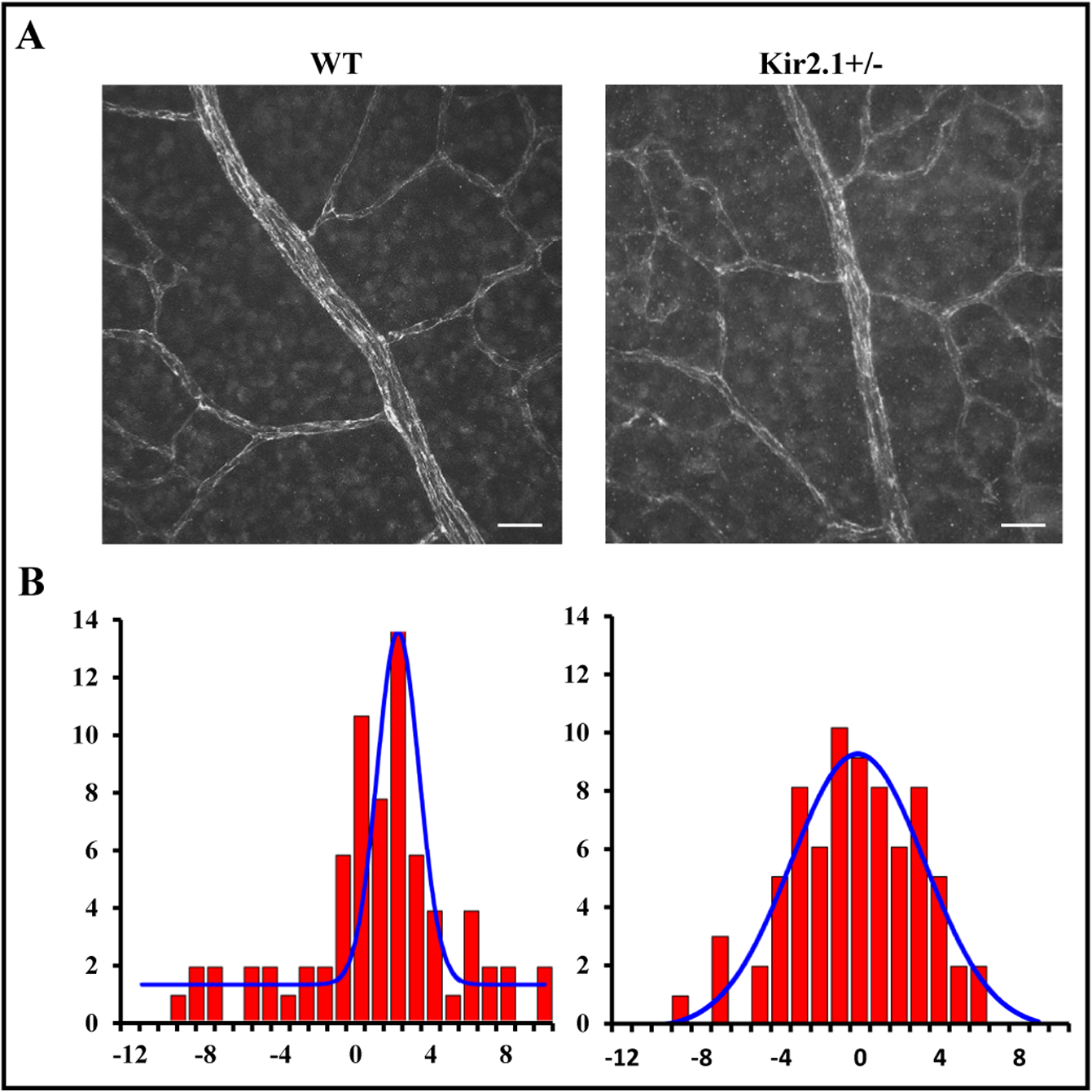
Kir2.1^+/−^ retinas show impaired endothelial cell alignment. Images of retina P6 stained for CD31 to visualize the contours endothelial cells. (B) Histograms of EC angles relative to the axis of the vessel determining the direction of the flow. Data are means ± SD of at least six mice per group. *P≤0.05. Scale bar panel A: 50 μm.

To address whether Kir2.1 channels play a role in shear stress mechanotransduction in the developing retina, we analyzed endothelial alignment, a hallmark of endothelial response to flow [5]. Earlier studies established that similarly to adult vasculature and to endothelial cultures *in vitro*, shear stress also induces endothelial alignment in the developing vessels of murine yolk sac [7]. To determine the role of Kir2.1 in endothelial alignment in the arteries of the developing retina, the whole-mounted retinas at P6 were stained for CD31, a major endothelial marker that is expressed on endothelial plasma membranes. Fig 5A shows that the contours of the cells and their orientation are clearly visible, both in larger and smaller arteries. Alignment was analyzed by measuring the angles between the long axis of individual cells and the direction of the vessel, which clearly represents the direction of flow in the vessel. All major vessels were analyzed in each retina. In this analysis, angle 0 indicates cells that align precisely in the direction of vessel (flow), whereas deviations from 0 indicate cells that align at an angle relative to the direction of the flow. Fig. 5B shows that Kir2.1 deficiency resulted in a significant broadening of the angle distribution indicating impaired alignment. The alignment was analyzed using “full width at half maximum” (FWHM), a parameter that is commonly used to describe the width of a function obtained for each of the Gaussian distributions fit to the data. The FWHM values are 2.7+0.5 and 7.9+0.8 for the WT and Kir2.1^+/−^ cells respectively. These data indicate that Kir2.1 plays a role in endothelial mechanotransduction in response to flow in the developing vasculature of the retina.

### Kir2.1 is critical for the cerebrovascular response to flow

**Fig. 6.**
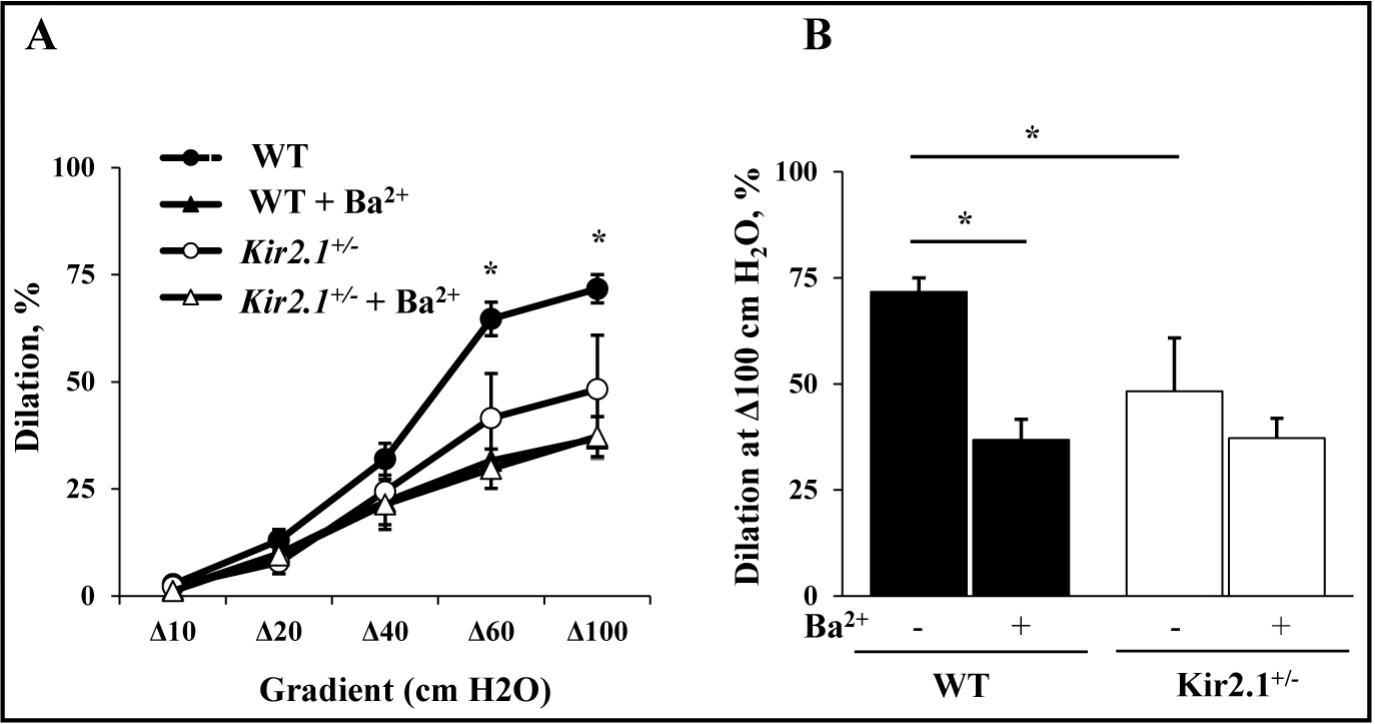
MCA Kir2.1 channels are critical components of FIV. A) FIV curves produced by exposing MCAs from WT and Kir2.1^+/−^ mice to intraluminal flow via the pressure gradient method. Vasodilation is shown as the percent dilation to baseline diameters recorded at physiological pressure (~44 mm Hg) after pre-constriction with ET-1 (120–200 pM) to ~50% of baseline. BaCl_2_ (30 μM) is used to block Kir channels and reduce FIV as previously reported [10]. B) Dilations to Δ100 cm H2O intraluminal flow reveal 1) significant inhibition of FIV by Ba^2+^ in WT MCA (*p<0.05; repeated measures 2-way ANOVA) and 2) a significant difference in FIV between WT and Kir2.1+/−(*p<0.05; 2 way-ANOVA). No effect of Ba^2+^ was observed in Kir2.1^+/−^ MCA.

To provide further evidence that Kir2.1 channels are a key element of endothelial shear stress-induced mechanotransduction in cerebral circulation, we tested the role of these channels in flow-induced vasodilation of cerebral circulation, another hallmark of endothelial response to flow. Since measuring flow-induced dilations of retinal arterioles is beyond our technical capabilities, these measurements were performed with cerebral resistance arteries. We previously established a role for Kir2.1 in endothelialdependent vasodilation to intraluminal flow, termed flow-induced vasodilation (FIV), in human and mouse arteries [10]. In order to test the role of Kir2.1 in acute vasoactive function to shear stress in cerebral arteries, middle cerebral arteries (MCA) were isolated from 20 week old WT or Kir2.1^+/−^ excised mouse brains and cannulated ex vivo to be visualized by video microscopy equipped to measure arterial diameters. After incubating at physiological pressures for one hour (60 cm H2O = ~44 mm Hg), baseline artery diameters were similar between groups (96.2 ± 10.4 μm for WT vs. 97.7 ± 19.3 μm for Kir2.1^+/−^). After pre-constriction with endothelin-1 (ET-1), arteries were exposed to increases in intraluminal flow via the pressure gradient method [44]. Fig. 6 shows a Ba^2+^-sensitive response to intraluminal flow in MCA from WT mice with dilations reaching ~71.7% the baseline diameter at Δ100 cm H2O. Incubation with BaCl_2_ (30 μM) reduces dilations to flow in WT MCA to ~36.8% (Δ100 cm H2O) of the baseline diameter. In contrast, dilations to flow are blunted in MCA from Kir2.1^+/−^ mice, reaching only 48.2% of the baseline diameter at Δ100 cm H2O. Furthermore, MCA from Kir2.1^+/−^ mice are no longer sensitive to Ba^2+^ indicating that Kir2.1 channels are required for the full FIV response in mouse MCA. These data verify further the critical role of Kir2.1 in endothelial mechanotransduction.

## Discussion

Formation of a functional and highly branched network of blood vessels is critical for tissue growth and function. One of the critical steps of blood vessel formation is sprouting angiogenesis which includes several steps: endothelial cell tip and stalk cell determination, proliferative vascular patterning, and finally a remodeling phase to provide enough blood flow to tissue. In the current study, we describe a novel mechanism that regulates angiogenesis in the developing retina via shear-stress sensitive inwardly-rectifying K^+^ channels, Kir2.1. Furthermore, we show more specifically that Kir2.1 is involved not in the sprouting stage of the angiogenesis but in pruning of the excess vessels and vascular remodeling. We propose that Kir2.1 is a critical component of a mechanosensory complex that at least partially mediates the effects of shear stress, a major factor in the regulation of angiogenesis.

During normal spouting angiogenesis, there is an initial excess of blood vessels that undergo partial degradation and remodeling as the retina matures. The key observation leading to the conclusion that Kir2.1 channels regulate the remodeling phase (pruning) of retinal angiogenesis is a significant decrease in the number of vessels undergoing degradation (“empty sleeves”) in Kir2.1^+/−^ retinas. Furthermore, the location within the vascular plexus that is affected by Kir2.1 deficiency corresponds to the region that undergoes the most pruning, as evidenced by the peak in the number of collagen-positive/isolectin negative “empty” sleeves. It is well known that in the mouse, growth of retinal vessels initiates after birth (P1) and expands from the center to retina edge during first week (P7) and that vessel sprouting is followed by remodeling and pruning. It is also known that in retinas of 6-day pups (P6) used in our study, the pruning is most prominent 50–80% of the distance from optic nerve to the edge of retina, the same region that we observe a significant difference between the WT and the Kir2.1^+/−^ retinas. A lack of an effect on the rate of cell proliferation and on filapodia is consistent with this conclusion.

Multiple studies showed that mechanical signals generated by blood flow are important factors in blood vessel development [5, 45]. However, while a variety of endothelial mechanosensing molecules have been discovered, including a variety of ion channels [46], junctional mechanosensitive complexes (VE-cadherin, Pecam-1,VEGFR) [47, 48], G-proteins [49], primary cilia [50, 51] and glycocalyx [52], the mechanisms by which shear stress regulates angiogenesis are still virtually unknown. In this study, we present several lines of evidence suggesting that Kir2.1 channels play an important role in shear stress-induced modulation of vascular development. Endothelial Kir channels have long been known to be one of the earliest endothelial responses to shear stress [25, 53] and were recently demonstrated by our group to be essential for flow-induced vasorelaxation of mesenteric arteries [10]. In the current study, we demonstrate that Kir2.1 are also required for flow-induced vasodilation of cerebral arteries. Kir2.1 deficiency also results in impaired endothelial alignment in the arteries of the developing retina further supporting their role in shear stress-induced mechanotransduction in cerebral and retinal vasculature. Most importantly, we show here that Kir2.1 regulates retinal angiogenesis via vessel regression while having no effect on endothelial proliferation and sprouting, the same regulatory mode that has been described previously for shear stress in angiogenesis in several developmental models [6–9]. These defects can also be inter-related, as a defect in the formation of the normal vasculature may result in the disrupted hemodynamic pattern of the shear stress forces, which in turn may manifest itself in a partial loss of endothelial alignment. Notably, this pattern of shear stress appears to be unique for *in vivo* angiogenesis, in microfluidics *in vitro* models, shear stress was shown both to induce and to inhibit endothelial sprouting [54, 55]. Clearly, while the *in vitro* microfluidics systems allow very sophisticated and well-controlled manipulation of the environment, they may fall short of recapitulating the complexity of the *in vivo* environment. Our study, therefore, focuses on retinal angiogenesis *in vivo*, one of the most powerful models for developmental angiogenesis [3], that also correlates with the mechanisms of angiogenesis in other organs [27, 32].

In terms of the mechanism, we propose that Kir2.1 contributes to vessel pruning via regulation of polarized endothelial migration induced by shear stress. Earlier studies showed that the mechanism by which shear stress regulates vessel regression and pruning in the retina is cell-death independent and is due to dynamic migration of endothelial cells from segments under low flow to segments under higher flow [8, 56]. In a developing retina this effect is expected to be observed most prominently in the middle of the distance from the optic nerve to the angiogenic front. This is exactly what we observed in this study in Kir2.1^+/−^ mice suggesting a delay in vasculature remodeling. Therefore, we propose that impairment of shear stress sensitivity of Kir2.1^+/−^ deficient endothelial cells compromises their ability to respond to the gradients of shear stress that drive this migration. More specifically, we suggest that endothelial cells in Kir2.1^+/−^ retinas are less sensitive to differential shear stress levels between bigger and smaller vessels and, thus, the movement of endothelial cells from segments under lower flow to higher flow is delayed. Mechanistically, an attractive hypothesis is that activation of Kir2.1 may facilitate endothelial migration via eNOS activation. We have recently shown that Kir2.1 channels are essential for flow-induced phosphorylation of eNOS and production of NO [10]. This was demonstrated using the same Kir2.1^+/−^ mouse model, as is implemented in the current study. Remarkably, an early study by Pipili-Synetos et al. [57] showed that inhibiting eNOS in-vivo with pan-NOS inhibitor l-NMMA results in an increase in vessel density in the chick embryo angiogenesis model, a similar effect to that observed in our current study in Kir2.1^+/−^ retinas. Interestingly, inhibition of eNOS was also shown to abrogate the effect of shear stress on endothelial migration *in vitro*, which became insensitive to shear stress in the presence of l-NMMA [55]. Another intriguing possibility is that activation of Kir channels may contribute to flow sensitivity of endothelial cells via augmenting endothelial hyperpolarization induced by Ca^2+^-sensitive K^+^ channels. Indeed, it was shown that Kir2.1 channels can be “boosters” of endothelial hyperpolarization mediated by Ca^2+^-sensitive K^+^ channels, as described by Goto et al. 2004 [58] and Sonkusare et al. 2016 [59]. It is also well-known that Ca^2+^-sensitive K^+^ channels play a major role in flow-induced vasodilation via NO-independent pathway, frequently termed EDH [10]. Further studies are needed to explore the possible roles of the Kir2.1/eNOS and Kir2.1/EDH pathways in angiogenesis.

Mural cells, pericytes and vSMCs also play a crucial role in the development of the blood vessels and promoting vascular quiescence through their interactions with endothelial cells. We observed no difference between WT and Kir2.1^+/−^ retinas in the pericytes coverage but found a significant decrease in vSMC coverage of the arteries. Interestingly, it was shown previously that shear stress enhances endothelial secretion of chemo-attractants that stimulate vSMCs migration and possibly EC-vSMCs interactions [60]. Thus, it is possible that a decrease in vSMCs coverage is also related to the impairment of endothelial shear stress signaling.

It is important to note, however, that we cannot rule out a possibility that the role of Kir channels in angiogenesis is independent of shear stress and might be mediated by more general effects of these channels on membrane potential and Ca^2+^ signaling in both endothelial cells and vSMCs. It is impossible to fully discriminate between these possibilities at the moment because the mechanism of shear stress sensitivity of Kir is poorly understood and there are no known Kir mutations that abrogate their sensitivity to shear. Further studies are needed to explore these mechanisms and to extend the current study to models of pathological retinal angiogenesis.

## Supplementary Material

Supplemental Material
