# Supplementary material for: Shear-Stress Sensitive Inwardly-Rectifying K+ Channels Regulate Developmental Retinal Angiogenesis by Vessel Regression
Source: Cell Physiol Biochem. Author manuscript; Available in PMC 2020 Mar 10. (PMC7063968; doi:10.33594/000000109)
Supplement: Supplemental Material [file NIHMS1067981-supplement-Supplemental_Material.pdf]

Supplemental Material

Shear-Stress Sensitive Inwardly-Rectifying K^+ Channels Regulate Developmental Retinal Angiogenesis by Vessel Regression

Evgenii Boriushkin[a] Ibra S. Fancher[b] Irena Levitan[b]

[a]Department of Medicine, Stony Brook University, Stony Brook, NY, USA, [b]Division of Pulmonary, Critical Care, Sleep and Allergy, Department of Medicine, University of Illinois at Chicago, Chicago, IL, USA

Supplement Figure 1

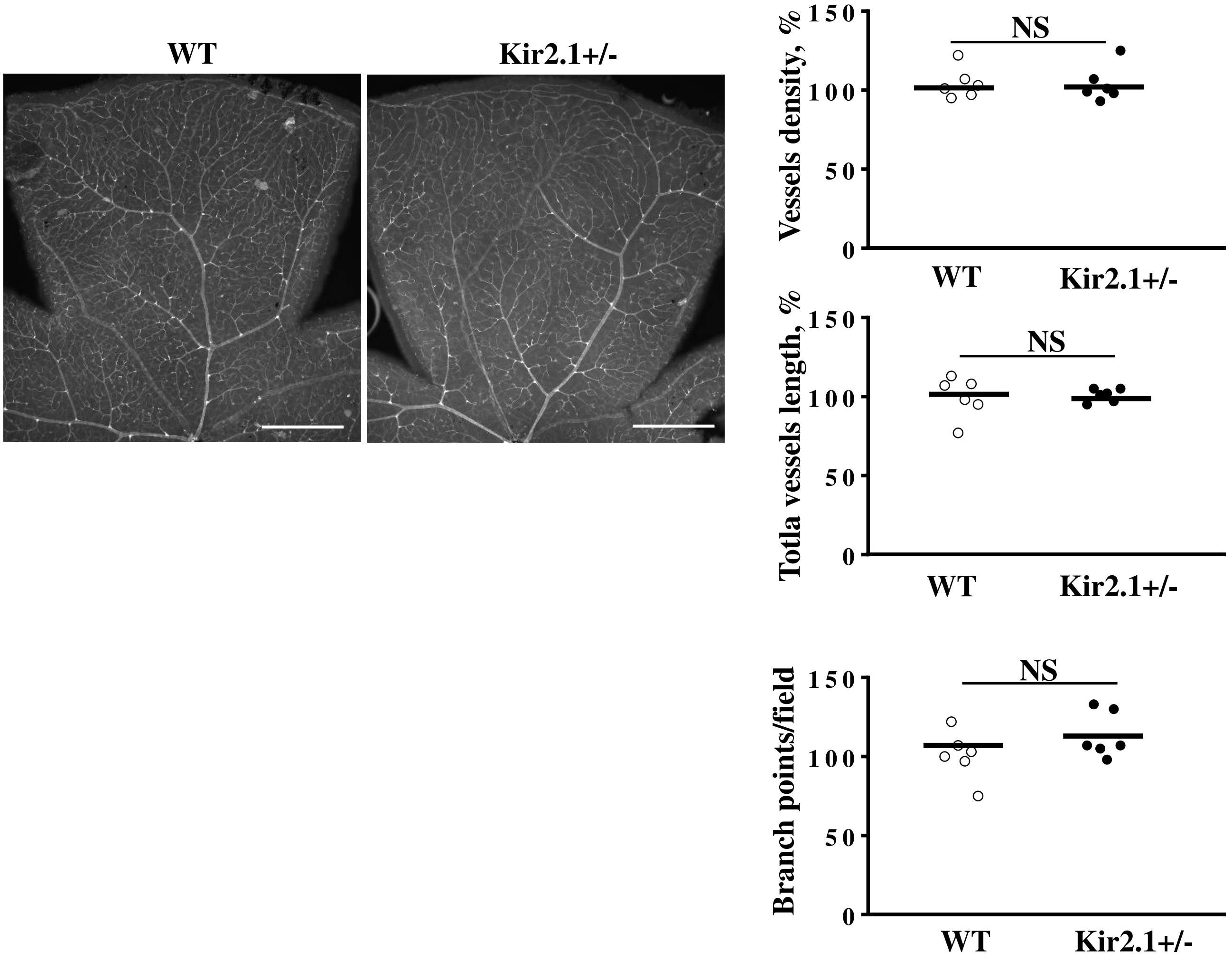

Supplemental figure 1. The analysis of retinal blood vessels in adult wild type and Kir2.1+/- mice showed no differences in vessels density, vessels length or branching. Data are means ± SD of six eyeballs per group. "NS" means no significant differences. Scale bar panel A: 100 μm.
